# Supplementary material for: Factors associated with changes in the objectively measured physical activity among Japanese adults: A longitudinal and dynamic panel data analysis
Source: PLoS One. 2023 Feb 16;18(2):e0280927. doi: 10.1371/journal.pone.0280927 (PMC9934362; doi:10.1371/journal.pone.0280927)
Supplement: S1 File — (DOCX) [file pone.0280927.s001.docx]

**Supporting information**

**Factors associated with changes in the objectively measured physical activity among Japanese adults: A longitudinal and dynamic panel data analysis**

Authors: Daiki Watanabe, Haruka Murakami, Yuko Gando, Ryoko Kawakami, Kumpei Tanisawa, Harumi Ohno, Kana Konishi, Azusa Sasaki, Akie Morishita, Nobuyuki Miyatake, and Motohiko Miyachi

**SUPPLEMENTARY TABLE LEGENDS**

**Table S1.** Number of individuals with information on all variables by in-person testing

**Table S2.** Assessment of multicollinearity for the multivariate panel data analysis

**Table S3.** Characteristics of participants included or excluded in this study

**Table S4.** Accuracy and precision of physical activity related variables in all participants

**Table S5.** Factors associated with changes of sedentary times by multivariate longitudinal analysis

**Table S6.** Sensitivity analyses for factors associated with changes of physical activity times by multivariate longitudinal analysis

**Table S7.** Sensitivity analyses for factors associated with changes of total energy expenditure, physical activity level, and step count by multivariate longitudinal analysis

**SUPPLEMENTARY FIGUREE LEGENDS**

**Figure S1.** Longitudinal trajectories of physical activity and total energy expenditure in 211 men (1089 measurements)

**Figure S2.** Longitudinal trajectories of physical activity and total energy expenditure in 478 women (2825 measurements)

**Table S1.** Number of individuals with information on all variables by in-person testing

|  | In-person testing (*n* = 689) [3914 measurements] | | | | | | | | | | | | |
| --- | --- | --- | --- | --- | --- | --- | --- | --- | --- | --- | --- | --- | --- |
|  | 1 | 2 | 3 | 5 | 6 | 7 | 8 | 9 | 10 | 11 | 12 | 13 | Total |
| Years of investigation | 2007 | 2008 | 2009 | 2010 | 2011 | 2012 | 2013 | 2014 | 2015 | 2016 | 2017 | 2018 |  |
| Age | 214 | 340 | 448 | 535 | 473 | 572 | 386 | 258 | 220 | 332 | 135 | 1 | 3914 |
| Area | 214 | 340 | 448 | 535 | 473 | 572 | 386 | 258 | 220 | 332 | 135 | 1 | 3914 |
| Sex | 214 | 340 | 448 | 535 | 473 | 572 | 386 | 258 | 220 | 332 | 135 | 1 | 3914 |
| Body Mass Index | 214 | 340 | 448 | 535 | 468 | 570 | 386 | 258 | 220 | 331 | 135 | 1 | 3906 |
| Waist/Hip ratio | 214 | 340 | 424 | 455 | 288 | 336 | 299 | 188 | 172 | 298 | 135 | 1 | 3150 |
| Comorbidity score ^a^ | 213 | 338 | 448 | 534 | 466 | 564 | 367 | 253 | 218 | 332 | 134 | 1 | 3868 |
| Smoking status | 214 | 340 | 448 | 535 | 473 | 572 | 386 | 258 | 220 | 332 | 135 | 1 | 3914 |
| Dietary survey | 195 | 339 | 448 | 533 | 463 | 570 | 386 | 257 | 220 | 331 | 135 | 1 | 3878 |
| Hemoglobin | 214 | 340 | 448 | 509 | 403 | 465 | 361 | 242 | 208 | 309 | 135 | 1 | 3635 |
| Heart rate | 214 | 340 | 448 | 456 | 288 | 336 | 301 | 188 | 172 | 298 | 135 | 1 | 3177 |
| Hand grips | 214 | 340 | 448 | 456 | 285 | 336 | 300 | 188 | 169 | 291 | 132 | 1 | 3160 |
| Leg power | 214 | 340 | 438 | 456 | 280 | 336 | 300 | 188 | 169 | 291 | 132 | 1 | 3145 |
| Trunk flexibility | 214 | 339 | 447 | 454 | 284 | 336 | 300 | 187 | 170 | 293 | 133 | 1 | 3158 |
| Physical activity | 214 | 340 | 448 | 535 | 473 | 572 | 386 | 258 | 220 | 332 | 135 | 1 | 3914 |
| Valid days of accelerometer data (day) |  |  |  |  |  |  |  |  |  |  |  |  | 3914 |
| Median | 14 | 19 | 20 | 23 | 23 | 23 | 22 | 22 | 23 | 23 | 25 | 14 |  |
| 25th percentile | 13 | 17 | 17 | 19 | 19 | 19 | 17 | 18 | 18 | 20 | 21 |  |  |
| 75th percentile | 14 | 20 | 25 | 27 | 27 | 27 | 26 | 26 | 26.8 | 27 | 28 |  |  |

Data for participants with missing values were imputed by multiple imputation: Body mass index (8 measurements), waist/hip ratio (764 measurements), comorbidity score (46 measurements), dietary survey (36 measurements), hemoglobin (279 measurements), heart rate (737 measurements), handgrip (754 measurements), leg power (769 measurements), and trunk flexibility (756 measurements).

^a^ Number of individuals which could obtain information on any of the 10 types of comorbidity status (including hypertension, dyslipidemia, diabetes, ischemic heart disease, other heart diseases, cerebrovascular diseases, renal failure, cancer, osteoporosis, and depression).

**Table S2.** Assessment of multicollinearity for the multivariate panel data analysis

|  | VIF |
| --- | --- |
| Age | 1.95 |
| Sex | 2.33 |
| Area | 1.72 |
| Body mass index | 1.66 |
| Waist/Hip ratio | 1.83 |
| Comorbidity score | 1.38 |
| Smoking status | 1.34 |
| Alcohol drinking status | 1.21 |
| Energy intake | 1.20 |
| Nutrient-Rich Food 9.3 score | 1.12 |
| Hemoglobin | 1.44 |
| Heart rate | 1.09 |
| Hand grips | 4.80 |
| Leg power | 3.92 |
| Trunk flexibility | 1.27 |

The values are shown as variance inflation factor (VIF).

**Table S3.** Characteristics of participants included or excluded in this study

|  | Included participants | | | Excluded participants | | | *p*-values |
| --- | --- | --- | --- | --- | --- | --- | --- |
|  | *n* | Distribution | | *n* | Distribution | |  |
| Age [years] ^a^ | 689 | 52.0 | (11.6) | 70 | 41.2 | (13.5) | <0.001 |
| Women [*n* (%)] ^b^ | 689 | 477 | (69.2) | 70 | 45 | (64.3) | 0.315 |
| Local area [*n* (%)] ^b^ | 689 | 227 | (32.9) | 71 | 29 | (40.9) | 0.180 |
| Body mass index [kg/m^2^] ^a^ | 689 | 22.5 | (2.9) | 70 | 22.2 | (3.2) | 0.486 |
| Waist/Hip ratio ^a^ | 689 | 0.88 | (0.07) | 70 | 0.84 | (0.06) | <0.001 |
| No comorbidity [*n* (%)] ^b^ | 689 | 524 | (76.1) | 70 | 56 | (78.9) | 0.377 |
| Smoker [*n* (%)] ^b^ | 689 | 196 | (28.4) | 70 | 18 | (25.4) | 0.581 |
| Alcohol drinker [*n* (%)] ^b^ | 689 | 501 | (72.7) | 70 | 54 | (76.1) | 0.546 |
| Energy intake [kcal/day] ^a^ | 689 | 1819 | (492) | 70 | 1727 | (533) | 0.140 |
| NRF 9.3 score ^a^ | 689 | 765 | (69) | 70 | 745 | (65) | 0.021 |
| Hemoglobin [g/dl] ^a^ | 689 | 13.6 | (1.4) | 70 | 13.7 | (1.5) | 0.775 |
| Heart rate [bpm] ^a^ | 689 | 63 | (12) | 690 | 65 | (12) | 0.176 |
| Hand grips [kg] ^a^ | 689 | 33.5 | (9.3) | 70 | 36.3 | (9.7) | 0.153 |
| Leg power [w] ^a^ | 689 | 1081 | (410) | 70 | 1222 | (411) | 0.006 |
| Trunk flexibility [cm] ^a^ | 689 | 39.1 | (9.8) | 70 | 41.3 | (11.2) | 0.081 |
| BMR [kcal/day] ^a^ | 689 | 1225 | (144) | 66 | 1292 | (157) | <0.001 |
| TEE [kcal/day] ^a^ | 689 | 1930 | (246) | 66 | 2017 | (301) | 0.006 |
| Physical activity level ^a^ | 689 | 1.58 | (0.14) | 66 | 1.56 | (0.13) | 0.121 |
| Step counts [steps/day] ^a^ | 689 | 10216 | (3556) | 66 | 9348 | (3087) | 0.048 |
| Inactive time [min/day] ^a^ | 689 | 1026 | (101) | 66 | 1052 | (96) | 0.042 |
| LPA [min/day] ^a^ | 689 | 354 | (94) | 66 | 331 | (93) | 0.049 |
| MVPA [min/day] ^a^ | 689 | 60 | (27) | 66 | 57 | (26) | 0.472 |

BMR, basal metabolic rate; LPA, low intensity physical activity; MVPA, moderate-to-vigorous physical activity; NRF, nutrient-rich food; TEE, total energy expenditure

^a^ Continuous variables were expressed as mean and standard deviation and were compared groups using the unpaired t-test.

^b^ Categorical variables were expressed as number and percentage and were compared groups using the chi-square test.

**Table S4.** Accuracy and precision of physical activity related variables in all participants

|  | Physical activity (*n* = 689) [3914 measurements] | | | | | |
| --- | --- | --- | --- | --- | --- | --- |
|  | Inactive time [min/day] | LPA  [min/day] | MVPA  [min/day] | Step [steps/day] | PAL | TEE  [kcal/day] |
| Mean (SD) at baseline | 1026 (101) | 354 (94) | 60 (27) | 10216 (3556) | 1.58 (0.14) | 1930 (246) |
| CV_w_ [%]^a^ | 4.1 | 10.8 | 22.6 | 17.0 | 4.0 | 4.5 |
| CV_b_ [%]^a^ | 10.3 | 26.4 | 49.6 | 36.9 | 9.3 | 14.9 |
| VR | 0.40 | 0.41 | 0.45 | 0.46 | 0.43 | 0.30 |
| ICC^b^ | 0.72 | 0.71 | 0.69 | 0.68 | 0.70 | 0.77 |
| Required group size^c^ |  |  |  |  |  |  |
| Specified % deviation |  |  |  |  |  |  |
| 1 | 475 | 3130 | 11414 | 6329 | 391 | 938 |
| 2.5 | 76 | 501 | 1826 | 1013 | 63 | 150 |
| 5 | 19 | 125 | 457 | 253 | 16 | 38 |
| 10 | 5 | 31 | 114 | 63 | 4 | 9 |
| Required survey periods^d^ |  |  |  |  |  |  |
| Specified correlation coefficient |  |  |  |  |  |  |
| 0.80 | 1 | 1 | 1 | 1 | 1 | 1 |
| 0.85 | 1 | 1 | 1 | 1 | 1 | 1 |
| 0.90 | 2 | 2 | 2 | 2 | 2 | 1 |
| 0.95 | 4 | 4 | 4 | 4 | 4 | 3 |
| Required survey periods^e^ |  |  |  |  |  |  |
| Specified % deviation |  |  |  |  |  |  |
| 2.5 | 10 | 71 | 313 | 177 | 10 | 13 |
| 5 | 3 | 18 | 78 | 44 | 2 | 3 |
| 10 | 1 | 4 | 20 | 11 | 1 | 1 |
| 20 | 0 | 1 | 5 | 3 | 0 | 0 |

CI, confidence interval; CV_w_, coefficient of within-person variation; CV_b_, coefficient of between-person variation; ICC, intraclass correlation coefficient; LPA, low intensity physical activity; MVPA, moderate-to-vigorous physical activity; PAL; physical activity level; r, correlation coefficient; SD, standard deviation; TEE, total energy expenditure; VR, within-person/between-person variance ratio

^a^ The CV_w_ and CV_b_ for physical activity related variables were calculated using analysis of variance.

^b^ ICC = CV_b_ / (CV_w_ + CV_b_). If the ICC is comparatively high, it means larger CV_b_ in physical activity related variables trajectory.

^c^ The group size = 1.96^2^ × [(CV_b_^2^ + CV_w_^2^)/D_0_^2^] required to estimate a group’s “true” mean physical activity related variables trajectory within a 95% CI with a specified % deviation (D_0_), where D_0_ is the specified % deviation. All values are group sizes.

^d^ The number of measurement times during study period (NT_1_) = [*r*^2^/(1 − *r*^2^)] × VR required to obtain a specified *r* between an individual’s measured value and unmeasured usual “true” mean physical activity related variables trajectory, where *r* is the specified correlation coefficient and an index of confidence related to an individual’s classiﬁcation or ranking within a population. All values are measurement times during study periods.

^|e^ The number of measurement times during study period (NT_2_) = (1.96 × CV_w_/D_1_)^2^ required to estimate an individual’s “true” mean physical activity related variables trajectory within a 95% CI with a specified % deviation (D_1_), where D_1_ is a specified % deviation. All values are measurement times during study periods.

**Table S5.** Factors associated with changes of sedentary times by multivariate longitudinal analysis

|  | Total  (*n* = 689 [3914 measurement]) | | Men  (*n* = 211 [1089 measurement]) | | Women  (*n* = 478 [2825 measurement]) | |
| --- | --- | --- | --- | --- | --- | --- |
|  | RC | 95% CI | RC | 95% CI | RC | 95% CI |
| Within R^2^ | *R^2^* = 0.003 | | *R^2^* = 0.004 | | *R^2^* = 0.004 | |
| Age [1 year] | -0.112 | (-0.358 to 0.133) | -0.067 | (-0.584 to 0.450) | -0.162 | (-0.440 to 0.115) |
| Women sex | 2.011 | (-5.953 to 9.975) |  | |  | |
| Local area | -1.046 | (-8.537 to 6.445) | 4.703 | (-9.025 to 18.432) | -4.722 | (-13.823 to 4.378) |
| BMI [1 kg/m^2^] | 0.915 | (0.024 to 1.806)* | 0.705 | (-1.240 to 2.651) | 0.997 | (0.007 to 1.987)* |
| Waist/Hip ratio [1 point] | -8.878 | (-33.346 to 15.590) | 9.205 | (-53.277 to 71.687) | -14.882 | (-40.529 to 10.764) |
| Comorbidity score [1 point] | 1.173 | (-0.650 to 2.997) | -0.387 | (-4.528 to 3.755) | 1.855 | (-0.129 to 3.840) |
| Smoker | 0.513 | (-4.346 to 5.373) | -0.495 | (-10.689 to 9.699) | 0.994 | (-4.417 to 6.406) |
| Alcohol intake [1 % energy] | 0.064 | (-0.219 to 0.347) | 0.341 | (-0.144 to 0.825) | -0.163 | (-0.523 to 0.198) |
| Energy intake [1 kcal/day] | 0.002 | (-0.001 to 0.005) | 0.002 | (-0.003 to 0.008) | 0.002 | (-0.001 to 0.006) |
| NRF 9.3 score [1 point] | 0.012 | (-0.009 to 0.033) | 0.006 | (-0.038 to 0.050) | 0.016 | (-0.008 to 0.039) |
| Hemoglobin [1 g/dl] | -0.071 | (-0.630 to 0.488) | -0.024 | (-2.761 to 2.712) | -0.146 | (-0.751 to 0.459) |
| HR [1 bpm] | 0.023 | (-0.102 to 0.149) | 0.113 | (-0.132 to 0.358) | -0.038 | (-0.185 to 0.108) |
| Hand grips [1 kg] | 0.190 | (-0.102 to 0.482) | 0.102 | (-0.445 to 0.648) | 0.226 | (-0.123 to 0.575) |
| Leg power [1 w] | -0.002 | (-0.008 to 0.004) | 0.000 | (-0.011 to 0.010) | -0.001 | (-0.009 to 0.007) |
| Trunk flexibility [1 cm] | -0.087 | (-0.238 to 0.063) | -0.065 | (-0.387 to 0.258) | -0.095 | (-0.264 to 0.074) |

BMI, body mass index; CI, confidence interval; HR, heart rate; LPA, low intensity physical activity; MVPA, moderate-to-vigorous physical activity; NRF, nutrient-rich food; RC, regression coefficients. The results of these analyses are expressed as RC with 95% CI. The RC and 95% CI were calculated for changes in physical activity-related variables per unit increment for covariates. Asterisk (*) indicates statistical significance (*p*<0.05). Sex and area were time-stable variables, and others were time-varying variables.

**Table S6**. Sensitivity analyses for factors associated with changes of physical activity times by multivariate longitudinal analysis

| Increment effects/unit | Physical activity times (*n* = 661 [3081 measurement]) | | | | | |
| --- | --- | --- | --- | --- | --- | --- |
|  | Inactive times | | LPA | | MVPA | |
|  | RC | 95% CI | RC | 95% CI | RC | 95% CI |
| Within R^2^ | *R^2^* = 0.016 | | *R^2^* = 0.009 | | *R^2^* = 0.026 | |
| Age [1 year] | -0.692 | (-1.194 to -0.188)* | 0.870 | (0.408 to 1.331)* | -0.178 | (-0.333 to -0.021)* |
| Women sex | -87.189 | (-105.693 to -68.684)* | 87.001 | (70.019 to 103.982)* | 0.149 | (-5.514 to 5.811) |
| Local area | -14.676 | (-30.801 to 1.449) | 28.925 | (14.123 to 43.725)* | -14.359 | (-19.248 to -9.469)* |
| BMI [1 kg/m^2^] | 4.934 | (2.935 to 6.932)* | -3.563 | (-5.396 to -1.729)* | -1.296 | (-1.916 to -0.675)* |
| Waist/Hip ratio [1 point] | 9.351 | (-48.793 to 67.494) | 0.850 | (-52.476 to 54.175) | -11.374 | (-29.951 to 7.202) |
| Comorbidity score [1 point] | 1.688 | (-1.977 to 5.354) | -0.128 | (-3.489 to 3.233)* | -1.658 | (-2.835 to -0.481)* |
| Smoker | 2.034 | (-7.649 to 11.717) | -0.716 | (-9.599 to 8.167) | -1.420 | (-4.466 to 1.626) |
| Alcohol intake [1 % energy] | -0.465 | (-1.045 to 0.115) | 0.172 | (-0.360 to 0.704) | 0.295 | (0.110 to 0.479)* |
| Energy intake [1 kcal/day] | -0.006 | (-0.011 to -0.001)* | 0.005 | (-0.001 to 0.009) | 0.001 | (-0.001 to 0.002) |
| NRF 9.3 score [1 point] | 0.000 | (-0.043 to 0.043) | -0.005 | (-0.044 to 0.035) | 0.004 | (-0.009 to 0.018) |
| Hemoglobin [1 g/dl] | 1.865 | (-0.790 to 4.520) | -1.872 | (-4.307 to 0.564) | -0.007 | (-0.858 to 0.844) |
| HR [1 bpm] | 0.436 | (0.172 to 0.699)* | -0.250 | (-0.491 to -0.008)* | -0.193 | (-0.277 to -0.108)* |
| Hand grips [1 kg] | 0.141 | (-0.722 to 1.003) | -0.336 | (-1.127 to 0.455) | 0.193 | (0.080 to 0.273)* |
| Leg power [1 w] | -0.013 | (-0.026 to 0.001) | 0.009 | (-0.004 to 0.021) | 0.004 | (0.000 to 0.008)* |
| Trunk flexibility [1 cm] | -0.649 | (-1.027 to -0.269)* | 0.275 | (-0.073 to 0.622) | 0.390 | (0.269 to 0.510)* |

BMI, body mass index; CI, confidence interval; HR, heart rate; LPA, low intensity physical activity; MVPA, moderate-to-vigorous physical activity; NRF, nutrient-rich food; RC, regression coefficients

The results of these analyses are expressed as RC with 95% CI. The RC and 95% CI were calculated for changes in physical activity-related variables per unit increment for covariates. Asterisk (*) indicates statistical significance (*p*<0.05). Sex and area were time-stable variables, and others were time-varying variables.

**Table S7**. Sensitivity analyses for factors associated with changes of total energy expenditure, physical activity level, and step count by multivariate longitudinal analysis

| Increment effects/unit | TEE | | PAL ^a^ | | Step counts | |
| --- | --- | --- | --- | --- | --- | --- |
|  | RC | 95% CI | RC | 95% CI | RC | 95% CI |
| N [measurement] | 661 [3081] | | 661 [3081] | | 661 [3081] | |
| Within R^2^ | *R^2^* = 0.022 | | *R^2^* = 0.020 | | *R^2^* = 0.086 | |
| Age [1 year] | -4.791 | (-5.904 to -3.678)* | 0.026 | (-0.051 to 0.103) | -79.915 | (-99.175 to -60.653)* |
| Women sex | -278.422 | (-318.478 to -238.365)* | 7.752 | (4.945 to 10.559)* | -379.126 | (-1078.221 to 319.969) |
| Local area | -29.714 | (-63.97 to 4.542) | -0.046 | (-2.467 to 2.374) | -2026.810 | (-2629.398 to -1424.222)* |
| BMI [1 kg/m^2^] | 17.973 | (13.531 to 22.414)* | -0.674 | (-0.981 to -0.365)* | -166.988 | (-243.761 to -90.214)* |
| Waist/Hip ratio [1 point] | -39.750 | (-176.914 to 97.413) | -8.363 | (-17.609 to 0.884) | -1741.695 | (-4052.003 to 568.613) |
| Comorbidity score [1 point] | -6.442 | (-15.174 to 2.290) | -0.446 | (-1.032 to 0.139) | -187.643 | (-334.099 to -41.185)* |
| Smoker | -0.864 | (-22.966 to 21.237) | -0.731 | (-2.245 to 0.782) | -66.546 | (-444.360 to 311.269) |
| Alcohol intake [1 % energy] | 1.760 | (0.407 to 3.112)* | 0.106 | (0.014 to 0.197)* | 45.821 | (22.910 to 68.730)* |
| Energy intake [1 kcal/day] | 0.015 | (0.001 to 0.029)* | 0.002 | (0.001 to 0.003)* | 0.078 | (-0.156 to 0.311) |
| NRF 9.3 score [1 point] | 0.023 | (-0.080 to 0.126) | 0.003 | (-0.004 to 0.009) | 0.787 | (-0.951 to 2.525) |
| Hemoglobin [1 g/dl] | -0.937 | (-7.244 to 5.369) | -0.198 | (-0.622 to 0.225) | -13.184 | (-119.112 to 92.745) |
| HR [1 bpm] | -1.226 | (-1.853 to -0.598)* | -0.100 | (-0.142 to -0.058)* | -17.786 | (-28.311 to -7.260)* |
| Hand grips [1 kg] | 2.100 | (0.102 to 4.097)* | -0.004 | (-0.140 to 0.131) | 41.058 | (7.139 to 74.976)* |
| Leg power [1 w] | 0.049 | (0.015 to 0.081)* | 0.002 | (-0.001 to 0.004) | 0.128 | (-0.562 to 0.818) |
| Trunk flexibility [1 cm] | 2.433 | (1.550 to 3.316)* | 0.135 | (0.075 to 0.194)* | 21.914 | (6.951 to 36.875)* |

BMI, body mass index; CI, confidence interval; HR, heart rate; NRF, nutrient-rich food; PAL; physical activity level; RC, regression coefficients; TEE, total energy expenditure

The results of these analyses are expressed as RC with 95% CI. The RC and 95% CI were calculated for changes in physical activity-related variables per unit increment for covariates. Asterisk (*) indicates statistical significance (*p*<0.05). Sex and area were time-stable variables, and others were time-varying variables.

^a^ The RC and 95% CI shown in the estimated value corrected by the 10^2^ because the estimated value was small.


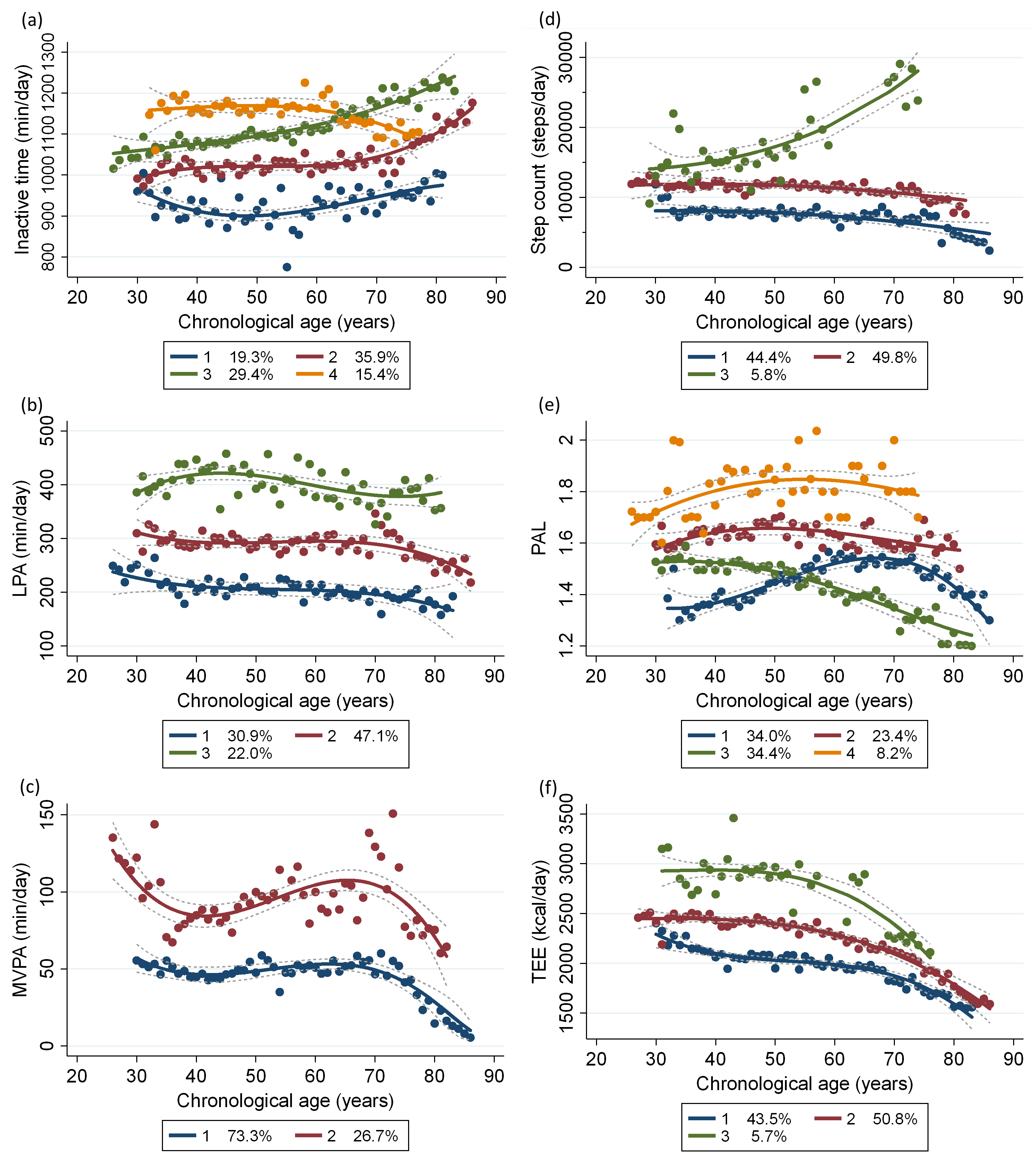


**Figure S1.** Longitudinal trajectories of physical activity and total energy expenditure in 211 men (1089 measurements)

The latent class growth models identified each distinct trajectory groups on (a) inactive time, (b) low-intensity physical activity (LPA), (c) moderate-to-vigorous physical activity (MVPA), (d) step count, (e) physical activity level (PAL), and (f) total energy expenditure (TEE) from ages 26 to 85 years through the maximum likelihood method. The solid lines represent group's mean physical activity related variables trajectory, and the dashed lines represent 95% confidence intervals.


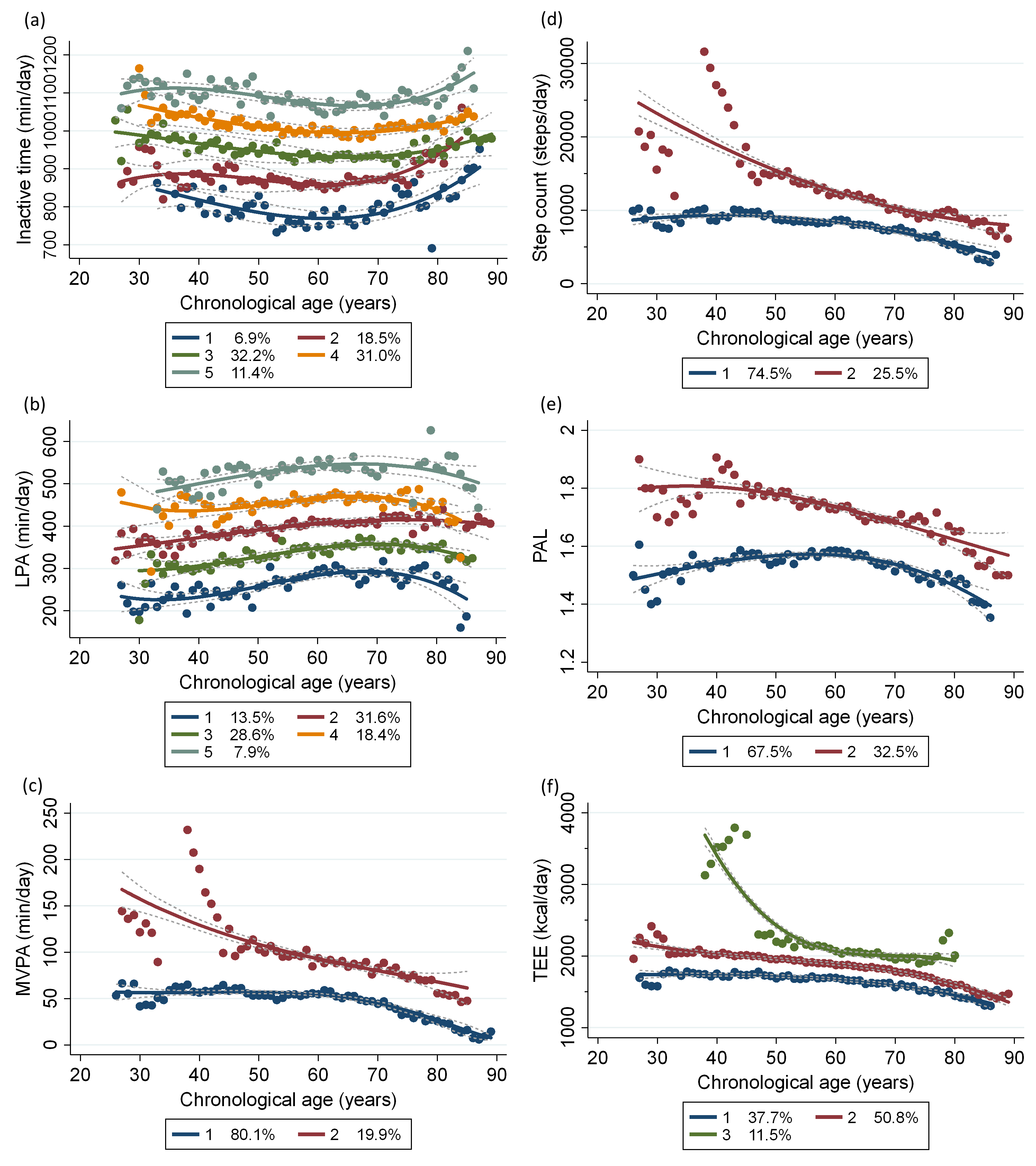


**Figure S2.** Longitudinal trajectories of physical activity and total energy expenditure in 478 women (2825 measurements)

The latent class growth models identified each distinct trajectory groups on (a) inactive time, (b) low-intensity physical activity (LPA), (c) moderate-to-vigorous physical activity (MVPA), (d) step count, (e) physical activity level (PAL), and (f) total energy expenditure (TEE) from ages 26 to 90 years through the maximum likelihood method. The solid lines represent group's mean physical activity related variables trajectory, and the dashed lines represent 95% confidence intervals.
